# Differences in the performance of adjuvant chemotherapy between hemodialysis and nonhemodialysis patients

**DOI:** 10.1002/cam4.5258

**Published:** 2022-09-21

**Authors:** Taisuke Ishii, Tomone Watanabe, Takahiro Higashi

**Affiliations:** ^1^ Division of Health Services Research National Cancer Center Tokyo Japan

**Keywords:** adjuvant chemotherapy, breast cancer, colon cancer, gastric cancer, hemodialysis, medical records, non‐small cell lung cancer, registries

## Abstract

**Background:**

The survival of hemodialysis (HD) patients with cancer is poor, which may be caused by undertreatment due to renal dysfunction. Particularly, adjuvant chemotherapy after surgery may be considered optional because of its preventive nature. This study investigated the current frequency of administration of adjuvant chemotherapy to HD patients compared with non‐HD patients in Japan.

**Methods:**

We used data from the Hospital‐Based Cancer Registries national database linked to health services utilization data to analyze cases of newly diagnosed colon cancer, gastric cancer, breast cancer, and non‐small cell lung cancer (NSCLC) at the stages where adjuvant chemotherapy is generally required. We compared the performance rate of adjuvant chemotherapy and the adjuvant chemotherapy regimens between HD and non‐HD patients from October 2011 to December 2017.

**Results:**

Of the 99,761 patients who underwent curative surgery, 1207 (1%) were HD patients. HD patients received adjuvant chemotherapy less frequently than non‐HD patients (24% vs. 63%, *p* < 0.001). After adjusting for potential confounders, HD remained negatively related to adjuvant chemotherapy administration for all four cancer types. Among all patients who received adjuvant chemotherapy 0(*N* = 61,873), HD patients were less likely to receive standard regimens and chemotherapy requiring dose adjustment than non‐HD patients (88% vs. 95%, *p* < 0.001 and 92% vs. 98%, *p* < 0.001, respectively). This trend was particularly pronounced among patients with gastric cancer.

**Conclusions:**

HD patients were less likely to receive adjuvant chemotherapy with standard regimens than non‐HD patients.

## LAY SUMMARY

Among patients newly diagnosed with colon cancer, gastric cancer, breast cancer, and non‐small cell lung cancer at the stages where adjuvant chemotherapy is generally required, adjuvant chemotherapy was less likely performed in hemodialysis (HD) patients than for non‐HD patients. HD patients tended not to receive the standard regimens compared to non‐HD patients partly due to the characteristics of each drug handling. Since the appropriate regimens for HD patients remain unclear, even in the adjuvant setting, future studies should evaluate the efficacy and safety of adjuvant chemotherapy and suggest appropriate adjuvant chemotherapy regimens for patients with HD.

PRECIS

Hemodialysis (HD) patients were less likely to receive adjuvant chemotherapy than non‐HD patients.

A larger proportion of non‐HD patients underwent standard chemotherapy regimens compared with HD patients.

Hemodialysis (HD) patients were less likely to receive adjuvant chemotherapy than non‐HD patients. This trend was consistent among the respective cancer types that we assessed: the odds of HD patients receiving adjuvant chemotherapy was approximately 0.1‐0.18 times less than non‐HD patients (colon cancer: OR = 0.15, 95% CI: 0.13‐0.19, P < 60; 0.001; gastric cancer: OR = 0.12, 95% CI: 0.09‐0.16, P < 60; 0.001; NSCLC: OR = 0.14, 95% CI: 0.09‐0.20, P < 60; 0.001; and breast cancer: OR = 0.17, 95% CI: 0.09‐0.31, P < 60; 0.001)

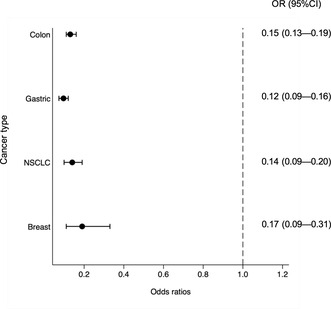

## 1 INTRODUCTION

The number of dialysis patients undergoing chronic hemodialysis (HD) treatment is increasing worldwide.^1^ Advances in dialysis therapy have improved the survival of HD patients, which has led to an increased risk of cancer in certain populations.^2^ It was reported that the risk of cancer is 1.44 times higher,^3^ and the risk of cancer mortality is 1.5–2.9 times higher in HD patients compared with the general population.^4^, ^5^, ^6^ Thus, the development of cancer treatments for HD patients is a crucial goal in oncology and nephrology fields.

Some specialists have suggested that HD patients receive insufficient cancer treatment, which would contribute to the high risk of cancer mortality among this population.^7^ However, evidence regarding chemotherapy undertreatment in HD patients compared with non‐HD patients is rare. A study in France reported that only 28% of HD patients with newly diagnosed cancer received chemotherapy, and among those who did, 72% required dose adjustment.^7^ A Japanese study showed that even fewer (15%) patients with cancer on HD received chemotherapy.^8^ However, these studies only reported the simple proportion of HD patients with cancer who were receiving chemotherapy treatment irrespective of the cancer stage, which included early‐stage patients who did not require adjuvant chemotherapy. Furthermore, non‐HD patients were not included as a reference. Therefore, these data only provide limited information about the extent of chemotherapy underuse among HD patients.

Unlike palliative chemotherapy, adjuvant chemotherapy aims to eradicate undetectable cancer cells and to prevent cancer recurrence. Thus, oncologists usually use well‐established chemotherapy regimens for indicated patients. Clinical trials have shown that adjuvant chemotherapy improved overall survival in many cancer types, including colon cancer, gastric cancer, non‐small cell lung cancer (NSCLC), and breast cancer.^9^, ^10^, ^11^, ^12^ However, these clinical trials excluded patients with significant renal dysfunction. Thus, it remains unclear whether the evidence obtained from clinical trials can be extrapolated to HD patients.

Although renal toxicity itself is unproblematic for HD patients, dose adjustment may be necessary to avoid severe side effects because the drugs used for chemotherapy or targeted therapy mainly undergo renal excretion.^2^, ^13^, ^14^, ^15^ However, in an adjuvant setting, where the choice of chemotherapy is usually more limited than palliative chemotherapy, the extent of the underuse of chemotherapeutic drugs that require dose adjustment remains unclear.

The provision of appropriate therapy is essential to reduce cancer mortality among HD patients. However, there is almost no high‐quality evidence that specifically targets HD patients, leaving the treatment decisions to the frontline clinicians and their patients. Although the level of evidence is not optimal, the practice of frontline clinicians may express their belief of what can be effective given the lack of sufficient evidence, forming collective wisdom that could provide some hints for potentially effective treatment options that could be further explored. Thus, we evaluated the rate of adjuvant chemotherapy administration to postoperative HD patients with cancer compared with postoperative non‐HD patients with cancer and the differences in adjuvant chemotherapy regimens between HD and non‐HD patients.

## 2 METHODS

### 2.1 Data sources and study approval

We used the data collected in our quality‐of‐care monitoring project for this study.^16^ Briefly, we collected health service utilization data from the Diagnosis Procedure Combination (DPC) survey that are equivalent to health insurance claims and linked them to the national database of Hospital‐Based Cancer Registries (HBCR). Although the DPC survey data are not used for reimbursement, they include details of all billable health services that are contained in fee‐for‐service insurance claims, including surgery, prescription drugs, and HD performance. As of 2012, the DPC database covered >50% of all beds in general hospitals in Japan.^17^ The HBCR is a compulsory cancer‐reporting system for all cancer care at key hospitals designated by the Ministry of Health, Labor, and Welfare in Japan.^18^ Additionally, nondesignated hospitals that have roles equal to the designated hospitals in their community optionally participate in the HBCR. The proportion of patients who were covered in the HBCR among all newly diagnosed cancer patients was 72.5% in 2018. About 61.2% of the hospitals included in the HBCR participated in the quality‐of‐care monitoring project. The HBCR has been operated following the national rule and records data on basic clinical information, including age at diagnosis, gender, cancer type, clinical and pathological cancer stages, tumor‐node‐metastasis classification, histopathology results coded according to the International Classification of Diseases for Oncology, 3rd edition, and the name of the treating hospital. We collected DPC survey data for patients registered in the HBCR from October of the previous year of diagnosis to the end of the following year of diagnosis. Consistent with the previous study,^16^ 94.8% of patients registered in HBCR were able to be linked to the DPC data in this study. We identified patients who underwent HD and received adjuvant chemotherapy using the linked DPC survey data.

This study was approved by the Institutional Review Board of the National Cancer Center, Japan (approval number 2020–195). It was conducted following the principles of the Declaration of Helsinki.

### 2.2 Study population

We examined data from adult patients with colon cancer, gastric cancer, NSCLC, and breast cancer who were newly diagnosed between January 2012 and December 2016 at hospitals that participated in our extensive quality‐of‐care monitoring in Japan.^19^ We evaluated the performance rate of adjuvant chemotherapy by focusing on patients who were followed up at least once after discharge by the hospital where they had undergone surgery and whose specific stage of postoperative cancer generally called for the recommendation of adjuvant chemotherapy (i.e. p‐stage [pathological stage] III colon cancer, p‐stage II–III gastric cancer [except for pT1 and pT3N0], p‐stage II–IIIA NSCLC, and p‐stage III breast cancer).^16^ We assessed the proportion of patients who received adjuvant chemotherapy and the types of regimens used. We excluded patients who received preoperative chemotherapy, those treated at hospitals where HD had never been performed, and those who received concurrent chemoradiation therapy.

### 2.3 Definitions

Based on a previous report, we defined adjuvant chemotherapy as postoperative chemotherapy initiated within 3 months after surgery for colon cancer and gastric cancer and postoperative chemotherapy initiated within 6 months after surgery for NSCLC and breast cancer.^16^ We identified patients undergoing HD using DPC survey data and defined those whose codes were related to HD services as HD patients. Because these definitions might have included temporary HD for acute kidney injury, we also performed a sensitivity analysis by redefining HD patients as those who received HD for more than 30 days. Based on clinical practice guidelines, we defined standard regimens as follows: 5‐fluorouracil (5‐FU) + oxaliplatin, 5‐FU, capecitabine + oxaliplatin, capecitabine, tegafur‐uracil (UFT), and tegafur‐gimeracil‐oteracil (S‐1) for colon cancer; S‐1, capecitabine + oxaliplatin, and 5‐FU for gastric cancer; cisplatin (CDDP) + pemetrexed (PEM), CDDP + gemcitabine (GEM), CDDP + docetaxel, CDDP + vinorelbine, CDDP + etoposide, carboplatin (CBDCA) + PEM, CBDCA + GEM, CBDCA + paclitaxel, and UFT for NSCLC; and doxorubicin or epirubicin + cyclophosphamide, taxane, doxorubicin or epirubicin + cyclophosphamide +5‐FU, docetaxel + cyclophosphamide, cyclophosphamide + methotrexate +5‐FU, and CBDCA + taxane for breast cancer.^9^, ^10^, ^11^, ^12^ Additionally, we used previous studies and Japanese guidelines^2^, ^13^, ^14^, ^15^ to identify chemotherapy regimens requiring dose adjustment for HD patients according to the first regimen administered after surgery. We defined the duration of adjuvant chemotherapy as the number of days from the initiation of postoperative chemotherapy to the end of chemotherapy that was sequentially administered within an interval of less than 100 days. The hospital type was classified as either a designated or nondesignated cancer care hospital, based on the designation status assigned by the Ministry of Health, Labour, and Welfare.

### 2.4 Statistical analysis

We compared the overall demographic characteristics of HD patients with those of non‐HD patients and within cancer types using Student's *t* test or the Mann‐Whitney U test for continuous variables and the chi‐square test for categorical variables. We used multiple logistic regression analyses adjusting for age at diagnosis, gender, and hospital type (designated vs. nondesignated) to evaluate whether HD was independently related to the administration of adjuvant chemotherapy. We used a stricter HD definition in the sensitivity analysis. Additionally, we compared the proportion of standard regimens and regimens requiring dose adjustment between HD and non‐HD patients who received adjuvant chemotherapy. Furthermore, we evaluated the frequency of standard regimens and chemotherapies requiring dose adjustment for each cancer type.

All statistical analyses were two‐sided and performed by using Stata v13.2 software (StataCorp LP, College Station, TX, USA). A *p*‐value <0.05 was considered statistically significant. GraphPad Prism 9 software (GraphPad Software Inc., San Diego, CA, USA) was used to construct bar graphs.

## 3 RESULTS

### 3.1 Patient characteristics

Our study cohort included 99,761 patients who underwent curative surgery for certain stages of colon cancer, gastric cancer, NSCLC, or breast cancer at 476 hospitals in Japan. Overall, 1207 (1%) were receiving HD. The HD patients were older than the non‐HD patients (mean age: 71 years vs. 69 years, *p* < 0.001) and more likely to be male (76% vs. 56%, *p* < 0.001; Table 1). The proportion of the four cancer types was highest for colon cancer and lowest for breast cancer among the HD patients (53% and 5%, respectively). The postoperative length of stay was approximately 8 days longer in HD patients compared with non‐HD patients (23 days vs. 15 days, *p* < 0.001).

**TABLE 1:** Patient characteristics stratified by dialysis

Characteristics	Total (*N* = 99,761)	HD patients (*n* = 1207)	Non‐HD patients (*n* = 98,554)	*p*‐value
Age in years, mean (SD, range) [years]	69 (11, 20–104)	71 (10, 26–94)	69 (11, 20–104)	<0.001
<65, *n* (%)	30,361 (30.4)	302 (25.0)	30,059 (30.5)	<0.001
65–75, *n* (%)	35,429 (35.5)	487 (40.4)	34,942 (35.5)	
>75, *n* (%)	33,971 (34.1)	418 (34.6)	33,553 (34.1)	
Gender (female), *n* (%)	43,321 (43.4)	295 (24.4)	43,026 (43.7)	<0.001
Cancer type, *n* (%)				<0.001
Colon cancer	47,174 (47.3)	635 (52.6)	46,539 (47.2)	
Gastric cancer	26,457 (26.5)	298 (24.7)	26,159 (26.5)	
NSCLC	17,864 (17.9)	212 (17.6)	17,652 (17.9)	
Breast cancer	8266 (8.3)	62 (5.1)	8204 (8.3)	
Duration of adjuvant chemotherapy, median (IQR) [days]	154 (98–259)	138 (35–182)	154 (98–260)	<0.001
Postoperative length of stay, median (IQR) [days]	15 (11–23)	23 (14–44)	15 (11–23)	<0.001
Hospital type, *n* (%)				0.62
Nondesignated hospital	13,078 (13.1)	164 (13.6)	12,914 (13.1)	
Designated hospital	86,683 (86.9)	1043 (86.4)	85,640 (86.9)	
Year of diagnosis, *n* (%)				0.78
2012	12,248 (12.3)	138 (11.4)	12,110 (12.3)	
2013	16,395 (16.4)	203 (16.8)	16,192 (16.4)	
2014	20,205 (20.3)	234 (19.4)	19,971 (20.3)	
2015	22,672 (22.7)	278 (23.0)	22,394 (22.7)	
2016	28,241 (28.3)	354 (29.3)	27,887 (28.3)	

Abbreviations: HD, hemodialysis; IQR, interquartile range; NSCLC, non‐small cell lung cancer; SD, standard deviation.

### 3.2 Performance rate and factors related to the performance of adjuvant chemotherapy

**FIGURE 1:**
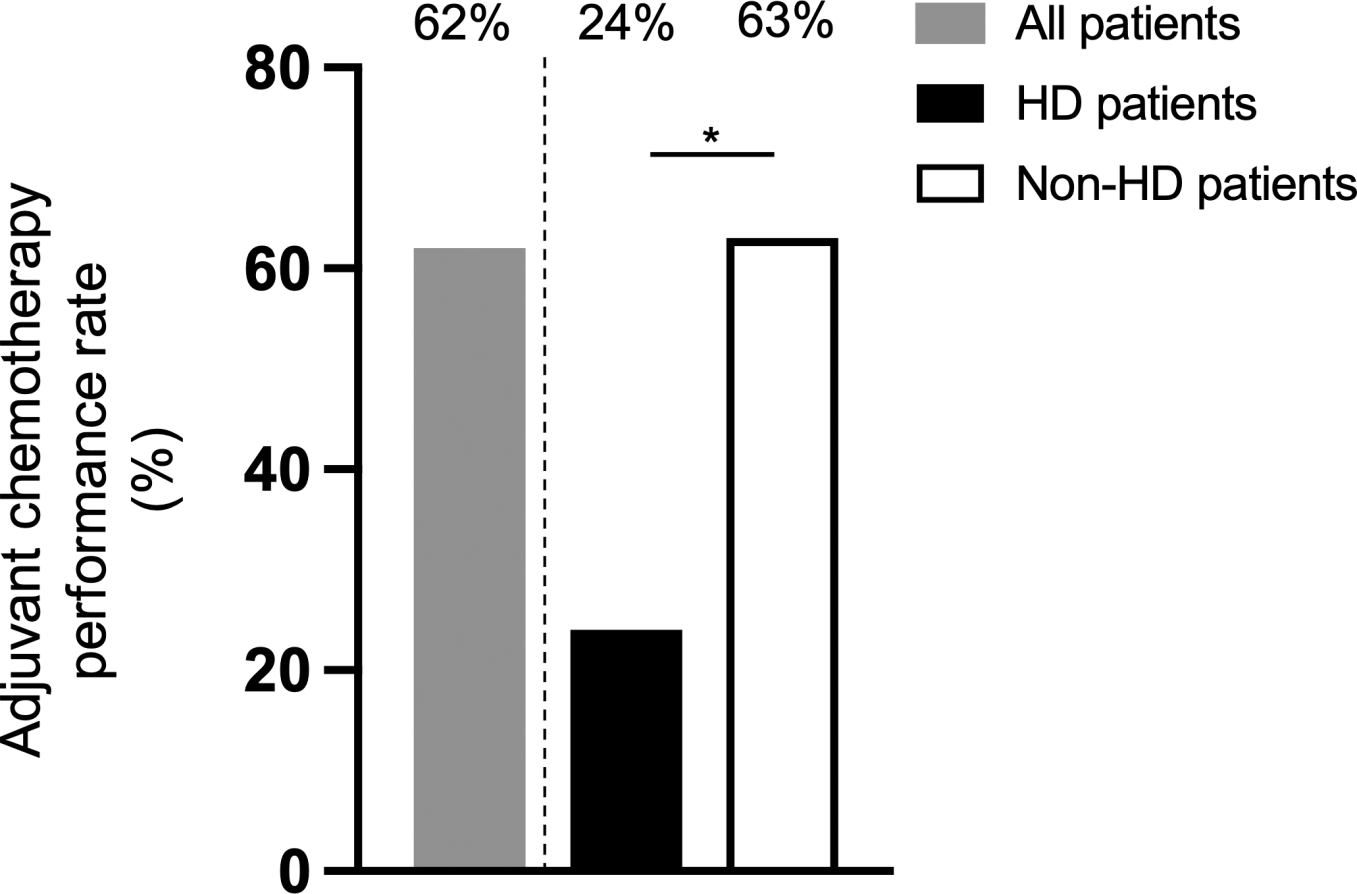
Differences in the administration of adjuvant chemotherapy between hemodialysis (HD) and non‐HD patients. Among all included patients (*N* = 99,761), 62.0% of patients received adjuvant chemotherapy. Additionally, HD patients received adjuvant chemotherapy less frequently than non‐HD patients (24% vs. 63%) *p < 0.001.

**FIGURE 2:**
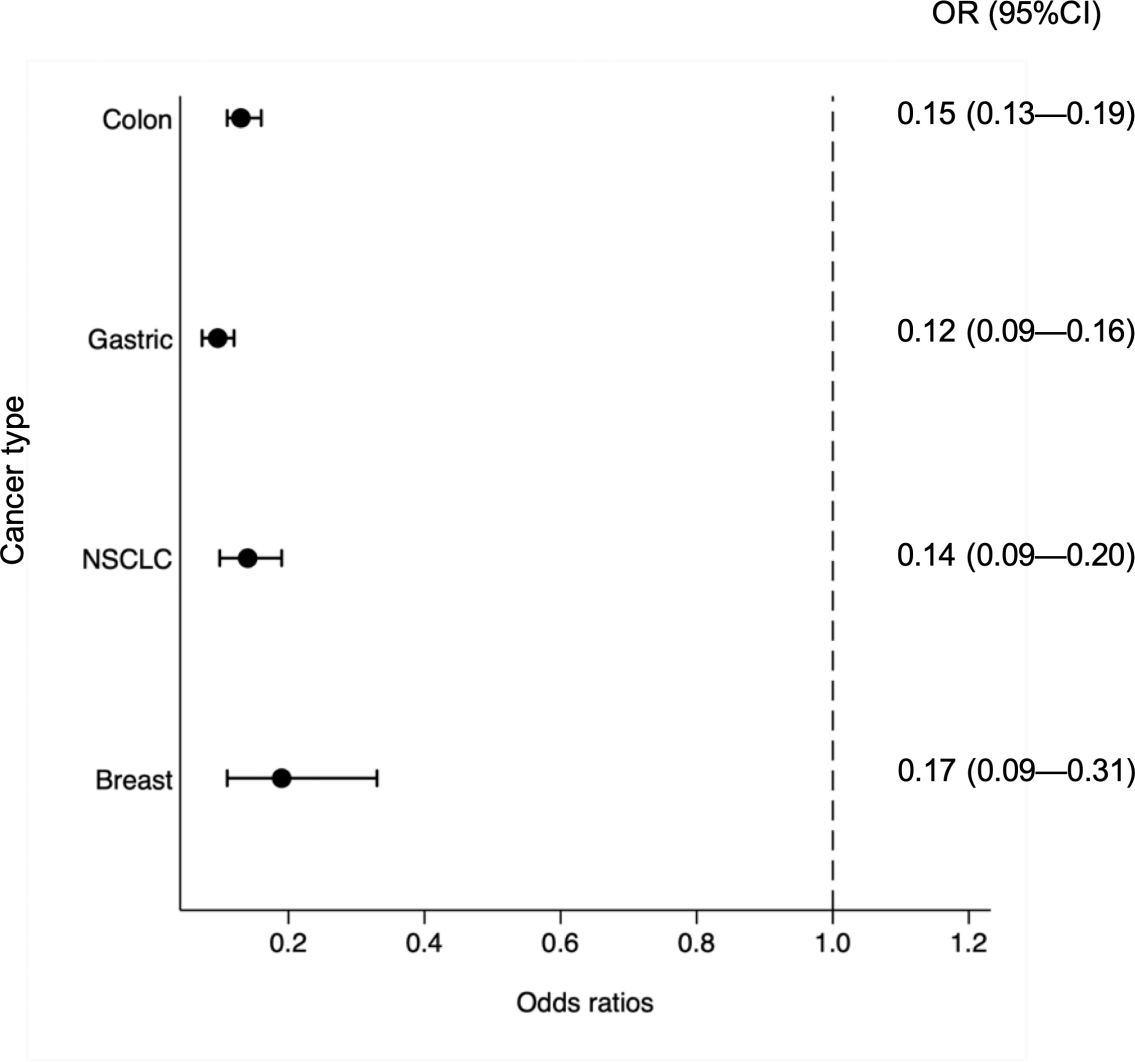
Adjusted OR of HD patients receiving adjuvant chemotherapy for each cancer type. For colon cancer, gastric cancer, NSCLC, and breast cancer, we set age at diagnosis, gender, and hospital type as covariates in logistic regression analysis for adjuvant chemotherapy performance. Among the respective cancer types, the odds of HD patients receiving adjuvant chemotherapy was approximately 0.1–0.18 times that of non‐HD patients (colon cancer: OR = 0.15, 95% CI: 0.13–0.19, *p* < 0.001; gastric cancer: OR = 0.12, 95% CI: 0.09–0.16, *p* < 0.001; NSCLC: OR = 0.14; 95% CI: 0.09–0.20, *p* < 0.001; and breast cancer: OR = 0.17; 95% CI: 0.09–0.31, *p* < 0.001), CI, confidence interval; NSCLC, non‐small cell lung cancer; OR, odds ratio.

HD patients received adjuvant chemotherapy less frequently than non‐HD patients (24% vs. 63%, *p* < 0.001; Figure 1). Moreover, among those who did receive chemotherapy, the median duration of adjuvant chemotherapy was significantly shorter among HD patients than among non‐HD patients (138 vs. 154 days, *p* < 0.001). After adjusting for age at diagnosis, gender, and hospital type, the overall odds of receiving adjuvant chemotherapy were significantly less among HD patients than among non‐HD patients. (odds ratio [OR] = 0.15, 95% confidence interval [CI]: 0.13–0.17; *p* < 0.001, Table 2). This trend was consistent among the respective cancer types that we assessed: the odds of HD patients receiving adjuvant chemotherapy was approximately 0.1–0.18 times less than non‐HD patients (colon cancer: OR = 0.15, 95% CI: 0.13–0.19, *p* < 0.001; gastric cancer: OR = 0.12, 95% CI: 0.09–0.16, *p* < 0.001; NSCLC: OR = 0.14, 95% CI: 0.09–0.20, *p* < 0.001; and breast cancer: OR = 0.17, 95% CI: 0.09–0.31, *p* < 0.001; Figure 2).

**TABLE 2:** Factors related to the performance of adjuvant chemotherapy for all patients with cancer (*N* = 99,761)

Characteristics	Unadjusted OR (95% CI)	*p* ‐value	Adjusted OR (95% CI)^a^	*p* ‐value
Dialysis				
No	Reference		Reference	
Yes	0.19 (0.16–0.21)	<0.001	0.15 (0.13–0.17)	<0.001
Age [years]				
<65	Reference		Reference	
65–75	0.59 (0.57–0.61)	<0.001	0.59 (0.57–0.62)	<0.001
>75	0.13 (0.12–0.14)	<0.001	0.13 (0.12–0.13)	<0.001
Gender				
Male	Reference		Reference	
Female	1.08 (1.05–1.11)	<0.001	1.09 (1.06–1.12)	<0.001
Hospital type				
Nondesignated	Reference		Reference	
Designated	1.06 (1.02–1.10)	0.002	0.96 (0.92–1.00)	0.05

Abbreviations: CI, confidence interval; OR, odds ratio.

^a^ Adjusted for age at diagnosis, gender, and hospital type.

### 3.3 Chemotherapy regimens

Among all patients who received adjuvant chemotherapy (*N* = 61,873), nonstandard regimens were more frequently administered to HD patients compared with non‐HD patients (12% vs. 5%, *p* < 0.001; Table 3). Moreover, chemotherapy regimens requiring dose adjustment were used less often for HD patients than for non‐HD patients (92% vs. 98%, *p* < 0.001; Table 4). In gastric cancer, the standard regimen was performed less often among HD patients than non‐HD patients (70% vs. 98%, *p* < 0.001; Table 3). Furthermore, HD patients infrequently received chemotherapy requiring dose adjustment compared with non‐HD patients in cases of colon cancer, gastric cancer, and NSCLC (95% vs. 99%, *p* < 0.001; 91% vs. 99%, *p* < 0.001; and 87% vs. 96%, *p* = 0.006, respectively; Table 4).

**TABLE 3:** Proportion of standard regimens among patients who received adjuvant chemotherapy

Characteristics	HD patients		Non‐HD patients		*p* ‐value
	*n*, (%)	Total	*n*, (%)	Total	
All patients	252 (88.4)	285	58,388 (94.8)	61,588	<0.001
Colon cancer	166 (97.6)	170	29,916 (98.4)	30,391	0.41
FOLFOX	15 (8.8)		2726 (9.0)		
Capecitabine + oxaliplatin	18 (10.6)		7906 (26.0)		
5‐FU	7–9 (4.1)		186 (0.61)		
Capecitabine	29 (17.1)		6833 (22.5)		
UFT	80 (47.1)		9463 (31.1)		
S‐1	17 (10.0)		2802 (9.2)		
Gastric cancer	47 (70.2)	67	16,429 (98.1)	16,744	<0.001
Capecitabine + oxaliplatin	0 (0)		477 (2.9)		
S‐1	46 (68.7)		15,914 (95.0)		
5‐FU	1–3 (1.5)		38 (0.23)		
NSCLC	22 (70.9)	31	7094 (77.6)	9138	0.37
Carboplatin‐based therapy	4–6 (19.4)		1957 (21.4)		
Cisplatin‐based therapy	13 (41.9)		4255 (46.6)		
UFT	4–6 (12.9)		882 (9.7)		
Breast cancer	17 (100)	17	4926 (92.7)	5315	0.26
AC, EC	11 (64.7)		1720 (32.4)		
Taxane	1–3 (17.7)		430 (8.1)		
CAF, FEC	1–3 (11.8)		2115 (39.8)		
TC	1–3 (5.9)		611 (11.5)		
CMF	0 (0)		36 (0.68)		
Carboplatin‐based therapy	0 (0)		14 (0.26)		

*Note*: The number of patients less than 10 is described in range due to the privacy protection rules of hospital‐based cancer registries.

Abbreviations: AC, doxorubicin + cyclophosphamide; CAF, doxorubicin + cyclophosphamide +5‐FU; CMF, cyclophosphamide + methotrexate +5‐FU; EC, epirubicin + cyclophosphamide; FEC, epirubicin + cyclophosphamide +5‐FU; FOLFOX, 5‐FU + leucovorin + oxaliplatin; 5‐FU, 5‐fluorouracil; HD, hemodialysis; NSCLC, non‐small cell lung cancer; TC, docetaxel + cyclophosphamide; UFT, tegafur‐uracil; S‐1, tegafur‐gimeracil‐oteracil.

**TABLE 4:** Proportion of HD patients who required dose adjustment among all patients who received adjuvant chemotherapy

Characteristics	HD patients		Non‐HD patients		*p* ‐value
	*n*, (%)	total	*n*, (%)	total	
All patients	263 (92.3)	285	60,319 (97.9)	61,588	<0.001
Colon cancer	161 (94.7)	170	30,105 (99.1)	30,391	<0.001
Gastric cancer	61 (91.0)	67	16,644 (99.4)	16,744	<0.001
NSCLC	27 (87.1)	31	8809 (96.4)	9138	0.006
Breast cancer	14 (82.4)	17	4717 (88.8)	5315	0.41

Abbreviations: HD, hemodialysis; NSCLC, non‐small cell lung cancer.

### 3.4 Sensitivity analysis

The use of a stricter definition of HD patients (i.e. patients who received HD for more than 30 days) did not change the results. Of all patients (*N* = 99,761), 565 (0.6%) received HD. HD patients received adjuvant chemotherapy less frequently than non‐HD patients (18% vs. 62%, *p* < 0.001; Supplementary Table S1). After adjusting for age at diagnosis, gender, and hospital type, HD remained an independent factor related to the performance of adjuvant chemotherapy (OR = 0.10, 95% CI: 0.08–0.13, *p* < 0.001; Supplementary Table S1).

## 4 DISCUSSION

Our findings revealed that HD patients with cancer received adjuvant chemotherapy far less often for colon cancer, gastric cancer, NSCLC, and breast cancer than non‐HD patients with cancer. Furthermore, even when HD patients did receive chemotherapy, the duration was often shorter, and the regimens were changed into nonstandard ones that did not require dose adjustment. The difference was particularly evident in gastric cancer patients. Ours is the first study that indicates the underuse of adjuvant chemotherapy for HD patients among some types of cancer. And this study could contribute to bringing awareness to the importance of future research in developing an optimal adjuvant treatment for HD patients.

Despite the importance of adjuvant chemotherapy to prevent recurrence after the surgical removal of tumors, the performance rate was significantly lower in HD patients than that in patients without HD for the indicated cancer and stages. Although previous reports suggested that the frequency of chemotherapy in HD patients was low in general, these studies did not reveal the full extent of chemotherapy underuse because they reported the overall chemotherapy performance rate and did not consider the cancer stage.^7^, ^8^ Our study was limited to patients where chemotherapy was indicated based on the clinical guidelines, and even when adjusted for age, gender, and the hospital type, the performance was low compared with non‐HD patients. This could be due to other preexisting comorbidities, postsurgical complications, or the physician's reluctance to administer chemotherapy because of the high rate of noncancer mortality among HD patients. Although there is little evidence demonstrating the efficacy of adjuvant chemotherapy for HD patients, it has been reported that anticancer therapy, including chemotherapy, improved cancer prognosis among HD patients.^20^ Therefore, adjuvant chemotherapy should be considered in a positive light for postoperative treatment of HD patients with cancer unless they have valid reasons that contraindicate chemotherapy.

Our study found that HD patients received standard regimens less often than non‐HD patients. Particularly, patients with gastric cancer were most affected by HD status, with a higher proportion of HD patients receiving nonstandard regimens compared with non‐HD patients. Of these nonstandard regimens that were administered to HD patients with gastric cancer, UFT was the most frequently used (50%; data not shown). UFT was previously used as standard adjuvant chemotherapy for gastric cancer in Japan,^21^ until S‐1 was proved more effective than the placebo in a large‐scale trial.^22^ Furthermore, S‐1 was more effective than UFT treatment.^23^ However, the Japanese guidelines do not currently recommend the use of S‐1 for HD patients because of concerns about excessive hematologic toxicity.^15^ Instead, dose‐adjusted UFT is allowed for treating HD patients. These facts seem to have affected the choice of adjuvant chemotherapy regimen for HD patients with gastric cancer in our study cohort. Thus, standard regimen for non‐HD patients is not always the most appropriate regimen for HD patients.

Based on clinical guidelines and previous studies, several cytotoxic chemotherapies and molecular‐targeted therapies require dose adjustment when used for HD patients.^2^, ^7^, ^13^, ^14^, ^15^ In our study, 92% of HD patients who received adjuvant chemotherapy required dose adjustment, which is much higher than the rate of 72% reported by Janus et al., who evaluated patients with all cancer stages, including stage IV.^7^ Compared with palliative chemotherapy, the choices of adjuvant chemotherapy are generally limited because the goal of adjuvant chemotherapy is to eradicate undetectable cancer cells, making it impossible to test nonstandard regimens and monitor the treatment response. Because we focused on adjuvant therapy, and most of these regimens for the four cancer types that we assessed (colon cancer, gastric cancer, NSCLC, and breast cancer) contained drugs that required dose adjustment for HD patients, our results showed a higher rate of chemotherapy using drugs requiring dose adjustment than in the previous study.

Our current study has some limitations. First, we did not have information concerning the patient's general condition, such as performance status and comorbidity. The prevalence of frailty among HD patients is higher than among non‐HD patients,^24^ which may be a reason for not administering chemotherapy. However, since our patients were all sufficiently fit to undergo curative surgery, preexisting comorbidities could not fully explain the large difference between HD and non‐HD patients. Surgical complications are another possible reason to refrain from chemotherapy because several studies reported a high complication rate among HD patients with lung cancer.^25^, ^26^, ^27^ In our study, the difference in median postoperative length of stay between HD and non‐HD patients was 8 days, which might have been caused by several postoperative complications. However, we defined adjuvant chemotherapy as postoperative chemotherapy started within 3 or 6 months after surgery. Therefore, it is unlikely that a delay in discharge of 8 days affected the decision to administer adjuvant chemotherapy in our study. Second, we were only able to assess data from hospitals that participated in our quality‐of‐care project. Thus, we were unable to detect the performance of adjuvant chemotherapy if it was administered at a dialysis clinic. However, in this current study, we focused on patients who were followed up by the hospital where they received surgery. Therefore, we believe that the number of patients who received chemotherapy at other hospitals will be negligible. Finally, we did not obtain data on patient survival. Thus, further studies are necessary to assess whether the low rate of adjuvant chemotherapy performance for HD patients affects mortality. To the best of our knowledge, this is the first study to show that standard regimens tend to not be used for HD patients. This finding might reflect the fact that standard regimens for the general population are not always appropriate for HD patients. Since adjuvant chemotherapy might be terminated earlier among HD patients than non‐HD patients due to adverse effects, the duration of adjuvant chemotherapy was shorter in HD patients in our current study. Thus, future studies should evaluate standard regimens and safety profiles for HD patients.

In conclusion, we used a nationwide database to show that HD patients were less likely to receive adjuvant chemotherapy than non‐HD patients and that HD was an independent factor related to adjuvant chemotherapy performance. Additionally, fewer HD patients were administered standard chemotherapy regimens and regimens requiring dose adjustment compared with non‐HD patients. The difference in adjuvant chemotherapy regimens between HD and non‐HD patients might be due to the characteristics of each drug handling. Unfortunately, the standard regimens for HD patients remain unclear, even in the adjuvant setting. Further studies are necessary to evaluate the efficacy and safety of adjuvant chemotherapy and to suggest standard adjuvant chemotherapy regimens for patients with HD.

## AUTHOR CONTRIBUTIONS

**Taisuke Ishii:** Conceptualization (lead); formal analysis (lead); writing – original draft (lead); writing – review and editing (equal). **Tomone Watanabe:** Data curation (equal); resources (equal); writing – review and editing (equal). **Takahiro Higashi:** Conceptualization (supporting); data curation (equal); funding acquisition (lead); resources (equal); supervision (lead); writing – review and editing (equal).

## FUNDING INFORMATION

This work was supported by a National Cancer Center Research and Development grant. [Grant number: H31‐A‐21].

## CONFLICT OF INTEREST

The authors have declared no conflicts of interest.

## ETHICS STATEMENT

This study was approved by the Institutional Review Board of the National Cancer Center, Japan (approval number 2020–195). It was conducted following the principles of the Declaration of Helsinki.

## Supporting information


Table S1
Table S2

## Data Availability

Data are used with special permission granted by privacy protection law and data use agreement with the hospitals. They are not publicly available.
