# Supplementary material for: Differences in the performance of adjuvant chemotherapy between hemodialysis and nonhemodialysis patients
Source: Cancer Med. 2022 Sep 21;12(4):4033–41. doi: 10.1002/cam4.5258 (PMC9972071; doi:10.1002/cam4.5258)
Supplement: Supplementary file 1 — Table S1 Table S2 [file CAM4-12-4033-s001.docx]

Table S1 Characteristics of patients stratified by dialysis that was defined as performance of dialysis for more than 30 days

| Characteristics | Total (N=99,761) | dialysis+ (N=565) | dialysis- (N=99,196) | *P*-value |
| --- | --- | --- | --- | --- |
| Age, mean (SD, min-max) [year] | 69 (11, 20-104) | 70 (9, 38-94) | 69 (11, 20-104) | 0.013 |
| <65, n (%) | 30,618 (29.8) | 146 (25.8) | 30,215 (30.5) | 0.016 |
| 65-75, n (%) | 36,128 (35.2) | 230 (40.7) | 35,199 (35.5) |  |
| 75<=, n (%) | 35,963 (35.0) | 189 (33.5) | 33,782 (34.1) |  |
| Gender (female), n (%) | 44,367 (43.2) | 140 (24.8) | 43,181 (43.5) | <0.001 |
| Cancer type, n (%) |  |  |  | 0.079 |
| Colon cancer | 48,567 (47.3) | 283 (50.1) | 46,891 (47.3) |  |
| Gastric cancer | 27,419 (26.7) | 141 (25.0) | 26,316 (26.5) |  |
| NSCLC | 18,365 (17.9) | 109 (19.3) | 17,755 (17.9) |  |
| Breast cancer | 8,358 (8.1) | 32 (5.7) | 8,234 (8.3) |  |
| Adjuvant chemotherapy, n (%) | 61,924 (60.3) | 99 (17.5) | 61,774 (62.3) | <0.001 |
| Duration of adjuvant chemotherapy, median (IQR) [days] | 154 (98-259) | 126 (14-189) | 154 (98-259) | <0.001 |
| Postoperative length of stay, median (IQR) [days] | 12 (9-17) | 15 (10-26) | 12 (9-17) | <0.001 |
| Hospital type, n (%) |  |  |  | 0.81 |
| Non-designated hospital | 13,533 (13.2) | 76 (13.5) | 13,002 (13.1) |  |
| Designated hospital | 89,176 (86.8) | 489 (86.6) | 86,194 (86.9) |  |
| Diagnosis year |  |  |  | 0.22 |
| 2012 | 12,633 (12.3) | 64 (11.3) | 12,184 (12.3) |  |
| 2013 | 16,875 (16.4) | 94 (16.6) | 16,301 (16.4) |  |
| 2014 | 20,818 (20.3) | 103 (18.2) | 20,102 (20.3) |  |
| 2015 | 23,316 (22.7) | 120 (21.2) | 22,552 (22.7) |  |
| 2016 | 29,067 (28.3) | 184 (32.6) | 28,057 (28.3) |  |

Abbreviations: SD, standard deviation; min, minimum; max, maximum; IQR, interquartile range; NSCLC, non-small cell lung cancer.

Table S2 Factors related to the performance of adjuvant chemotherapy for all cancer patients in sensitivity analysis where dialysis was defined as performance of hemodialysis for more than 30 days(N=99,761)

| Characteristics | Unadjusted Odds ratio (95% CI) | *P*-value | Adjusted Odds ratio (95% CI) | *P*-value |
| --- | --- | --- | --- | --- |
| Dialysis |  |  |  |  |
| No | Reference |  | Reference |  |
| Yes | 0.13 (0.10-0.16) | <0.001 | 0.10 (0.08-0.13) | <0.001 |
| Age [year] |  |  |  |  |
| <65 | Reference |  | Reference |  |
| 65-75 | 0.59 (0.57-0.61) | <0.001 | 0.59 (0.57-0.62) | <0.001 |
| 75<= | 0.13 (0.12-0.14) | <0.001 | 0.13 (0.12-0.13) | <0.001 |
| Gender |  |  |  |  |
| Male | Reference |  | Reference |  |
| Female | 1.08 (1.05-1.11) | <0.001 | 1.10 (1.07-1.13) | <0.001 |
| Hospital type |  |  |  |  |
| Non-designated | Reference |  | Reference |  |
| Designated | 1.06 (1.02-1.10) | 0.002 | 0.96 (0.92-1.00) | 0.06 |

Adjusted for Age, Gender, Hospital type, Dialysis

Abbreviations: CI, confidence interval.
